# Determinants of community acquired pneumonia among 2 to 59 months of age children in Northeast Ethiopia: a case-control study

**DOI:** 10.1186/s41479-020-00077-0

**Published:** 2020-12-05

**Authors:** Getaw Walle Bazie, Nebat Seid, Bitiya Admassu

**Affiliations:** 1 Department of Epidemiology and Biostatistics, School of Public Health, College of Medicine and Health Sciences, Wollo University, Dessie, Ethiopia; 2 Out Patient Department of Gerado Health Center, Dessie City Health Department, Dessie, Ethiopia; 3 Department of Population and Family Health, Faculty of Public Health, Jimma University, Jimma, Ethiopia

**Keywords:** Determinants, Community-acquired, Pneumonia, Under-five children, Ethiopia

## Abstract

**Background:**

Pneumonia is the leading cause of mortality and morbidity in under-five children. Regardless of this fact, efforts to identify determinants of pneumonia have been limited in the study area. The aim of this study was to identify determinants of community-acquired pneumonia among 2–59 months of age children in Northeast Ethiopia.

**Methods:**

Facility-based unmatched case-control study was conducted from February to April, 2019 among 444 (148 cases and 296 controls) 2–59 months of age children in Northeast Ethiopia. Cases were children with pneumonia, while controls were non-pneumonia children. Data were collected using a structured and pre-tested questionnaire by integrated management of neonatal and childhood illness trained nurses. The data were entered into Epi Data and then transferred to SPSS version 23 for analysis. Binary logistic regression analysis was used to test associations between the independent and the dependent variables. Variables with P-value ≤ 0.05 in the multivariable logistic regression model were declared as significant variables.

**Results:**

Children having older age mother (AOR = 0.03, 95% CI; 0.01,0.14), having mothers who are housewife (AOR = 0.19, 95% CI; 0.07,0.54), not having separate kitchen (AOR = 5.37; 95% CI: 1.65,17.43), having a history of diarrhea in the last 2 weeks (AOR = 10.2; 95% CI: 5.13, 20.18), having a history of acute lower respiratory infection in the last 2 weeks (AOR = 8.3, 95% CI: 3.32, 20.55) and having a history of parental asthma in the family (AOR = 4.9, 95% CI: 2.42, 10.18) were found to be determinants of community-acquired pneumonia.

**Conclusions:**

Children having older age mother, having mothers who are housewife, not having separate kitchen, having a history of diarrhea in the last 2 weeks, having a history of acute lower respiratory infection in the last 2 weeks and having a history of parental asthma in the family were found to be determinants of community-acquired pneumonia. Therefore, all health institutions should promote early treatments and prevention of diarrhea and acute lower respiratory infections of under-five children at the health facility and household level.

## Background

Community-acquired pneumonia (CAP) is an infection that begins outside the hospital or is diagnosed within 48 hours after admission to the hospital in a person who has not resided in a long-term care facility for 14 days or more before admission. Community-acquired pneumonia (CAP) is an infective inflammation of lung parenchyma due to bacterial or viral pathogens [1].

The incidence of pneumonia in children under the age of five years is 0.29 episodes per child-year, which equates 151.8 million cases annually in developing countries [2]. Childhood pneumonia remains a leading killer of children in developing countries where it accounts for up to 21% of deaths in children under the age of five years [3]. The mortality rates of children under the age of five years in most developing countries range from 60 to 100 per 1000 live births, one-fifth of these deaths are due to pneumonia [4].

In sub-Saharan Africa, the estimated proportion of death in children aged below 5 years attributed to pneumonia is 17–26% [5]. Ethiopia is the fifth (62 deaths in 1000) among 15 countries having the highest death rate of under five years of clinical pneumonia in the world [6, 7].

The determinants of pneumonia are numerous; educational status of parents, smoking habits of any member of the household, nutritional status, age and sex of the child and widely varies across the regions of the world [8]. Mortality due to childhood pneumonia is strongly linked to poverty-related factors such as undernutrition, lack of safe water and sanitation, indoor air pollution and inadequate access to health care. Around half of childhood pneumonia deaths are associated with air pollution. The effects of indoor air pollution kill more children globally than outdoor air pollution [9, 10]. Several risk factors for acquiring respiratory infections in developing countries, such as poverty, low family income, low parental education level, low birth weight, malnutrition, and lack of breastfeeding, have been described [11].

The rate of all-cause mortality in the under-five age group has been cut by more than half worldwide since 1990, from 91 deaths per 1000 live births to 43 in 2015. Although this is an enormous achievement, pneumonia and diarrhea’s contribution to under-five children death remains stubbornly high. In 2015, these two diseases together were responsible for nearly one of every four deaths that occurred in children under five [12–30].

The under-five pneumonia morbidity burden also costs the health services program as health services are passed on to cure high pneumonia morbidity cases. Pneumonia is not only the problem of individuals, but it is also equally the problem of policymakers, planners and communities at large. Controlling the continued threat of pneumonia is one of the major health priorities of the government of Ethiopia for which this study would contribute its part. The result would be used to ensure the continuity of continuum of care so that healthy preschool children will be transformed into healthy adolescents. More importantly, there were no previous scientific studies to find out the determinants of CAP among 2–59 months of age children in this study area though the recent service report from the study area found pneumonia to be one of the ten top diagnoses in children. Therefore, this study was intended to fill this information gap by identifying the determinants of CAP among 2 to 59 months of age children and to update the previous knowledge on the same problem.

## Methods and materials

### Study area and design

A facility-based unmatched case-control study was conducted from February 4 to April 15, 2019 among 2–59 months of age children in Northeast Ethiopia. The study was conducted in Dessie City, Northeast Ethiopia. It is located 401 KMs from Addis Ababa, the capital city of Ethiopia. It was conducted from February 4 to April 15, 2019.

### Population

The study population was children aged two months to five years who were attending Dessie city health institutions for different reasons within the data collection period and who lived in Dessie city administration kebeles for a minimum of six months. The cases were children aged two months to five years positive for pneumonia as defined by the World Health Organization (WHO) Integrated Management of Childhood Illness (IMNCI) guideline adopted by the Ethiopian Government since 2001 [10]. The controls were children aged two months to five years who have no pneumonia presented for immunization and growth monitoring and for other health services like Vitamin A supplementation and de-worming. Mothers/caretakers who had children 2–59 months of age come to health institution for health care services were included in the study. Mothers/caretakers who had children 2–59 months of age but severely ill and unable to communicate were excluded from the study.

### Variables

The dependent variable was community acquired pneumonia. The independent variables were socio-demographic variables (age, sex, occupation, educational status, place of residence and number of under-five children in the house hold), home based variables (housing condition, source of water, family smoking history and place of cooking), nutritional variables (nutritional status of child and breast feeding status), common childhood illnesses and related care practices (URTI, diarrhea, family pneumonia in the last two weeks, zinc supplementation and immunization status).

### Sample size and sampling procedures

The sample size was determined based on sample size calculation for two population proportions formula using EPI info version 7 software by calculating the minimum number of cases and controls required by taking assumptions of a 95 percent confidence level, 80 percent power, and 14.3 percent controls the history of current parental smoking giving odds ratio (OR) of 2.0 [31]. Finally, the total sample size was 444 (148 cases and 296 controls) by taking 1:2 case to control ratio.

In Dessie city administration, there are 8 health Centers; all of them were included in the study. Based on the number of clients/patients who visited each health center during the previous year, the total sample size was proportionally allocated to each selected health center. Screening for pneumonia in under-five outpatient department (OPD) and the maternal health department was performed based on the Integrated Management of Newborn and Child Illness (IMNCI) guideline. Controls were selected from growth monitoring and expanded program of immunization (EPI) units. The number of study participants was assigned to each selected health facility proportional to their average client size attended per month by referring to their monthly reports.

**Fig. 1:**
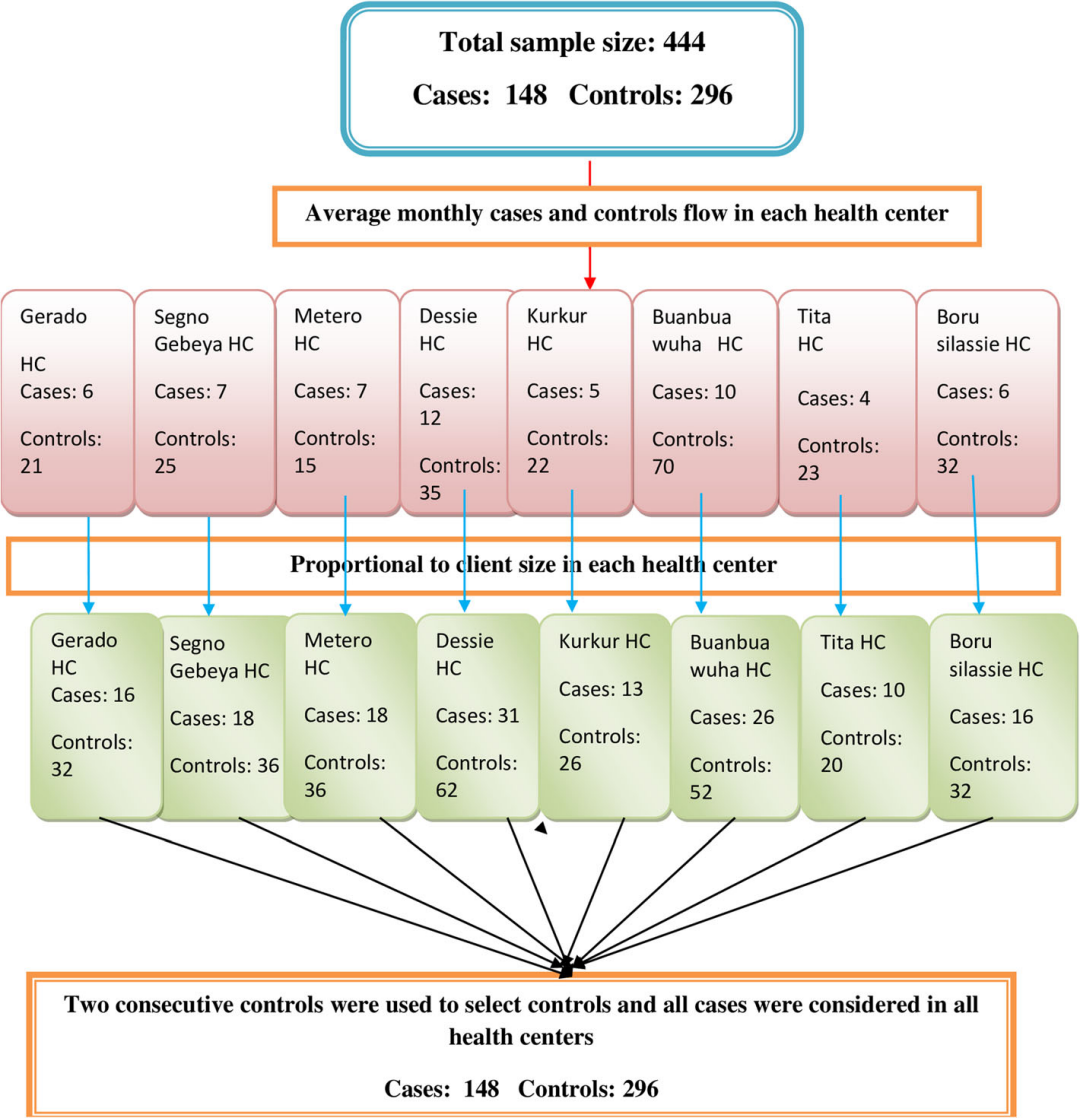
Sampling procedures of children 2–59 months of age in Northeast Ethiopia, 2019

The total yearly cases and controls were reported monthly through HMIS. Each health center’s report was divided by 12 to get the average monthly flow. To determine the sample size, required from each health center, the average monthly flow of cases and controls is multiplied by the duration of the study period (2 months and 1 week). Finally, the study subjects were drawn from each selected health facility using consecutive sampling. The youngest child at the time of the interview was included for mothers/caretakers who have more than one under-five children. All cases were considered in all health centers during the study period and for each case, two consecutive controls were used to select controls (Fig. 1).

### Data collection instruments and procedures

Data were collected by IMNCI trained nurses working in under-five clinics using a structured and pre-tested questionnaire. Study subjects were interviewed based on an interviewer-administered structured questionnaire. Eight nursing graduated data collectors and four experienced supervisors were recruited for the data collection. The questionnaire was first prepared in English. The English version questionnaire was translated to the Amharic version and was retranslated back to English to check its consistency. The questionnaire was on the possible determinants of CAP including socio-demographic factors, home-based factors, child’s nutritional status, childhood illnesses, and care practices. Finally, a record review was done to collect information on height, weight and zinc supplementation. The data quality was assured by properly designing and pre-testing the questionnaire, proper training of the interviewers and supervisors, proper categorization and coding of the questions. The one-day training was given to data collectors and supervisors before the initiation of the data collection. The questionnaire was pretested on 5% of the total sample out of the study area which has similar socio-demographic characteristics with the study health centers. Questionnaires were reviewed and checked for completeness by the supervisors and principal investigator and the necessary feedback was provided to data collectors in the next morning before the actual procedures begin.

### Data processing and analysis

After data collection, each questionnaire was checked visually for completeness. The corresponding code number was written carefully at each margin. Data cleaning was performed to check for accuracy, consistency and missing values and variables. The template scheme for data entry was developed and pre-tested for ranges, skipping patterns and allowed legal values by entering 5% pre-test questionnaires. The cleaned data were entered into Epi-Data version 3.1 and was exported to SPSS version 23 statistical software packages for further analysis. Descriptive statistics like frequency tables, percentages and figures were used to describe the study population in relation to other variables. Any errors identified at this stage were corrected by reviewing the original data using the code numbers. The bi-variable analysis was conducted primarily to identify individual variable which has an association with the dependent variable. Variables which have an association with the dependent variables at *P*-value ≤ 0.2 was a candidate in the multivariable logistic regression. The backward stepwise regression method was used to select the variables. Variables with *p*-values ≤ 0.05 were used to identify independent factors of CAP. The degree of association between independent and dependent variables was assessed using crude and adjusted odds ratios, and their statistical significance was assessed at a 95% confidence interval. Adequacy of the model was assessed using Hosmer and Lemeshow goodness of fit to test whether the required assumptions for the application of multivariable logistic regression are fulfilled.

### Ethical considerations

Ethical clearance was obtained from Institutional Review board of College of Medicine and Health Sciences, Wollo University. Permission letter to conduct the study was obtained from the city Health Department. Written informed consent was obtained from the children’s parents/guardians after they were informed about objectives and procedures of the study. Their rights to refuse participation any time they want were assured. For this purpose, a one-page consent letter was attached as a cover page of each questionnaire stating about the general objective of the study and issues of confidentiality which was discussed by the data collectors before proceeding with the interview. At the end of each interview, children who were cases were advised to follow their treatment.

## Results

### Socio-demographic characteristics

Overall, 444 child population (148 cases and 296 controls) were enrolled in the study making a 100% response rate for both study groups. Of these enrolled, 105 (70.9%) cases and 235 (79.4%) controls were permanent urban residents. Female children account for 70 (47.3%) of cases and 155 (52.4%) of controls. The mean (± SD) age of the children was 24.16 (± 14.10) months and 23.94 (± 15.30) months for cases and controls, respectively. The mean (± SD) age of mothers was 29.19 (± 4.18) years and 25.77 (± 5.06) years for cases and controls, respectively. In relation to the household monthly income of the respondents, a large proportion of mothers of cases reported a household monthly income 103 (69.59%) while mothers of controls reported 213 (72.0%) per month. The number of children greater than three was 113 (76.4%) of the cases and 207 (69.9%) controls were lived in the households (Table 1).

**Table 1:** Socio-demographic characteristics of children 2–59 months of age in Northeast Ethiopia, 2019

Variables	Pneumonia status	
	**Cases, n (%)**	**Controls, n (%)**
**Residence**		
Urban	105 (70.9)	235 (79.4)
Rural	43 (29.1)	61 (20.6)
**Sex of child**		
Male	78 (52.7)	141 (47.6)
Female	70 (47.3)	155 (52.4)
**Age of the child, months**		
2–11	31 (20.9)	81 (27.4)
12–23	46 (31.1)	85 (28.7)
24–35	32 (21.7)	51 (17.2)
36–47	24 (16)0.2	39 (13.2)
48–59	15 (10.1)	40 (13.5)
**Age of the mother, years**		
18–24	19 (12.8)	133 (44.9)
25–34	117 (79.1)	149 (50.4)
≥35	12 (8.1)	14 (4.7)
**Educational status of mother**		
Primary 1–4	32 (21.6)	58 (19.6)
Primary5-8	55 (37.1)	57 (19.2)
Secondary 9–12	47 (31.8)	108 (36.5)
College/higher education	14 (9.5)	73 (24.7)
**Marital status of mother**		
Married	130 (87.8)	283 (95.6)
Divorced /widowed	18 (12.2)	13 (4.4)
**Educational status of father**		
Non formal education	1 (0.7)	6 (2.0)
Primary 1–4	25 (16.9)	46 (15.5)
Primary 5–8	54 (36.5)	79 (26.7)
Secondary 9–12	36 (24.3)	52 (17.6)
College/higher education	32 (21.6)	113 (38.2)
**Mother’s occupation**		
Housewife	96 (64.9)	148 (50.0)
Student	16 (10.8)	38 (12.8)
Government employee	18 (12.2)	76 (25.7)
Merchant	12 (8.1)	26 (8.8)
Others^a^	6 (4.0)	8 (2.7)
**Household monthly income**		
< 2500 Birr	16 (10.82)	42 (14.1)
2500–6000 Birr	103 (69.59)	213 (72.0)
> 6000 Birr	29 (19.59)	41 (13.9)
**Number of under-five children in the family**		
1–2 children	35 (23.6)	89 (30.1)
> 3 children	113 (76.4)	207 (69.9)
Others^a^= refers to private employee, non-governmental organization employee and daily laborer		

### Home-based characteristics

Regarding the house status, 99.1% had a roof with corrugated iron sheets for both study groups. About 88 (59.5%) cases and 149 (50.3%) controls had an earthen floor and their wall was made of wood with mud. One hundred forty-three (96.6%) of cases and 294 (99.3%) of controls houses had windows. About 54.3% of children’s households used wood and dung as fuel for cooking. About 73 (49.3%) of cases and 169 (57.1%) of controls children were carried on the back during cooking. One hundred fifteen (77.7%) of cases and 210 (70.9%) of controls were cared for by their parents. Regarding the sleeping room, less than three persons in the family share bed during sleeping for cases 136 (91.9%) and controls 285 (96.3%) (Table 2).

**Table 2:** Home based characteristics of children 2–59 months of age in Northeast Ethiopia, 2019

Variables	Pneumonia status	
	**Cases, n (%)**	**Controls, n (%)**
**Main material of roof**		
Thatched roof	2 (1.4)	2 (0.7)
Corrugated iron	146 (98.6)	294 (99.3)
**Main material of floor**		
Earth with mud/others	88 (59.5)	149 (50.3)
Cement/brick	60 (40.5)	147 (49.7)
**Distance from Health Centers**		
< 5 km	86 (58.1)	53 (17.9)
5–10 km	50 (33.8)	174 (58.8)
>11 km	12 (8.1)	69 (23.3)
**Presence of Separate kitchen in the house**		
Yes	126 (85.1)	288 (97.3)
No	22 (14.9)	8 (2.7)
**Separate Cattle room**		
Yes	36 (24.3)	39 (13.2)
No	112 (75.7)	257 (86.8)
**Presence of window in the house**		
Yes	143 (96.6)	294 (99.3)
No	5 (3.4)	2 (0.7)
**Cooking practices in the houses**		
Wood/dung	75 (50.7)	166 (56.1)
Charcoal	10 (6.8)	20 (6.7)
Stove/gasoil	10 (6.8)	10 (3.4)
Electricity	53 (35.7)	100 (33.8)
**Mother/caregiver carrying child at the back while cooking**		
Yes	73 (49.3)	169 (57.1)
No	75 (50.7)	127 (42.9)
**Child caring practice in the house**		
Parental care	115 (77.7)	210 (70.9)
Housemaid	33 (22.3)	86 (29.1)
**Persons share bed during sleeping**		
Three or less	136 (91.9)	285 (96.3)
More than Three	12 (8.1)	11 (3.7)

### Nutritional characteristics

Regarding zinc supplementation during diarrhea, 119 (80.4%) of cases and 274 (92.6%) of controls had never been supplemented with zinc during diarrhea. About 99.5% of under-five children had been exclusively breastfed. One hundred thirty-four (90.5%) of cases and 278 (93.9%) of controls started complementary feeding at six months. About 3.7% of cases and 3.7% of controls were stunted and 2% of cases and 3.4 percent of controls were wasted (Table 3).

**Table 3:** Nutritional characteristics of children 2–59 months of age in Northeast Ethiopia, 2019

Variables	Pneumonia status	
	**Cases, n (%)**	**Controls, n (%)**
**Zinc Supplementation**		
Yes	29 (19.6)	22 (7.4)
No	119 (80.4)	274 (92.6)
**Current breast-feeding Status**		
Yes	84 (56.8)	160 (54.1)
No	64 (43.2)	136 (45.9)
**Birth to 6 months of breast feeding**		
Exclusive breast feeding	145 (97.9)	294 (99.3)
Non – Exclusive breast feeding	3 (2.1)	2 (0.7)
**Duration of breast feeding**		
No BF	4 (2.7)	7 (2.4)
12 months or less	71 (47.9)	89 (30.1)
13–24 months	63 (42.6)	175 (59.1)
>24 months	10 (6.8)	25 (8.4)
**Begin of complementary feeding**		
Before 6 months	14 (9.5)	18 (6.1)
After 6 months	134 (90.5)	278 (93.9)
**Nutritional status of the child**		
**Height for Age (HFA)**		
Not stunting	143 (96.6)	285 (96.3)
Stunting	5 (3.4)	11 (3.7)
**Weight for Age (WFA)**		
Normal	145 (98.0)	283 (95.6)
Underweight	3 (2.0)	13(4.4)
**Weight for Height (WFH)**		
Not Wasting	145 (98.0)	2861(96.6)
Wasting	3 (2.0)	10 (3.4)
**MUAC, centimeter**		
< 11	49 (33.1)	80 (27.0)
11–12	48 (32.4)	97 (32.8)
>12	51 (34.5)	119 (40.2)

N.B.: *MUAC* Middle upper arm circumference

### Common childhood illnesses and related care practices

Thirty-six (24.3%) of cases and 24.3% of controls had current diarrhea illness and two cases had a history of measles illness. About 118 (79.7%) of cases and 233 (78.7%) of controls received a full dose of pentavalent vaccine. One hundred twenty-two cases and 241 controls received measles vaccines. One hundred forty-five cases (98%) and 277 controls (93.6%) received one full dose of the pneumococcal conjugate vaccine (Table 4).

**Table 4:** Common childhood illnesses and related care practices of children 2–59 months of age in Northeast Ethiopia, 2019

Variables	Pneumonia status	
	**Cases, n (%)**	**Controls, n (%)**
**Diarrhea in the last 2 weeks**		
Yes	54 (36.5)	37 (12.5)
No	94 (63.5)	259 (87.5)
**Current diarrhea illness**		
Yes	36 (24.3)	72 (24.3)
No	112 (75.7)	224 (75.7)
**URTI in the last 2 weeks**		
Yes	28 (18.9)	75 (25.3)
No	120 (81.1)	221 (74.7)
**History of measles illness**		
Yes	1 (0.7)	1 (0.3)
No	147 (99.3)	295 (99.7)
**History of lower respiratory tract infection in the last 2 weeks**		
Yes	31 (20.9)	12 (4.1)
No	117 (79.1)	284 (95.9)
**History of parental asthma in the family**		
Yes	38 (25.7)	26 (8.8)
No	110 (74.3)	270 (91.2)
**A child received pentavalent vaccine**		
No vaccinated	2 (1.4)	1 (0.3)
1–2 dose taken	28 (18.9)	62 (20.9)
Full dose taken	118 (79.7)	233 (78.7)
**A child received measles vaccine**		
Yes	122 (82.4)	241 (81.4)
No	26 (17.6)	55 (18.6)
**A child received PCV vaccine**		
Yes	145 (98.0)	277 (93.6)
No	3 (2.0)	19 (6.4)

N.B.:* URTI *Upper respiratory tract infection, *PCV *Pneumococcal Vaccine

### Determinants of community-acquired pneumonia

After controlling for confounders on multivariable logistic regression, children having older age mother, having mothers who are housewife, not having separate kitchen, having a history of diarrhea in the last 2 weeks, having a history of acute lower respiratory infection in the last 2 weeks and having a history of parental asthma in the family were found to be determinants of community-acquired pneumonia.

The finding revealed that a child born from a mother whose age was between 18 and 24 years were 97% less likely to develop pneumonia (AOR = 0.03, 95% CI: 0.01, 0.14) compared to a child born to a mother whose age was 35 years or above. Children born from mothers who were government employees were 81% less likely to develop childhood community-acquired pneumonia (AOR = 0.19, 95% CI: 0.07, 0.54) compared to children born from mothers who worked as a housewife. Children from those households who had no separate kitchen for cooking were 5.4 times more likely to develop childhood community-acquired pneumonia (AOR = 5.4; 95% CI: 1.65,17.43) compared to children from households who had separation of kitchen from the main house.

Children who had a history of diarrhea in the past fifteen days prior to the study were ten times (AOR = 10.2; 95% CI: 5.13, 20.18) more likely to develop pneumonia compared to their counterparts. Children from households with a history of acute lower respiratory infection within the past fifteen days prior to the study were 8.3 times (AOR = 8.3, 95% CI: 3.32, 20.60) more likely to develop pneumonia compared to their counterparts. Likewise, children from households with a history of asthma were 4.9 times more likely (AOR 4.9, 95% CI 2.42, 10.18) to develop pneumonia compared to their counterparts (Table 5).

**Table 5:** Determinants of community-acquired pneumonia among children 2–59 months of age in Northeast Ethiopia, 2019

**Variables**	Pneumonia status			
	**Cases, n (%)**	**Controls, n (%)**	**COR (95% CI)**	**AOR (95% CI)**
**Age of the Mother, years**				
18–24	19 (12.8%)	133 (44.9%)	0.17 (0.07, 0.41) *	0.03 (0.01, 0.14) *
25–34	117 (79.1%)	149 (50.4%)	0.92 (0.41, 2.06)	0.51 (0.16, 1.62)
>=35	12 (8.1%)	14 (4.7%)	1.0	1.0
**Mother’s occupation**				
Housewife	96 (64.9%)	148 (50.0%)	1.0	1.0
Student	16 (10.8%)	38 (12.8%)	0.65 (0.34,1.23)	2.03 (0.73, 5.64)
Government employee	18 (12.2%)	76 (25.7%)	0.37 (0.21,0.65) *	0.19(0.07,0.54) *
Merchant	12 (8.0%)	26 (8.8%)	0.71 (0.34,1.48)	0.96 (0.37,2.52)
Others^**^	6 (4.1%)	8 (2.7%)	1.16 (0.39,3.44)	2.15 (0.47, 9.68)
**Separate kitchen with available windows during cooking**				
No	54 (36.5)	10 (3.4)	16.4 (8.05, 33.55) **	5.37 (1.65,17.43) **
Yes	94 (63.5)	286 (96.6)	1.0	1.0
**Diarrhea in the last 2 weeks**				
Yes	54 (36.5%)	37 (12.5%)	4.02 (2.49,6.50) **	10.2 (5.13,20.18) **
No	94 (63.5%)	259 (87.5%)	1.0	1.0
**History of lower respiratory tract infection in the last 2 weeks**				
Yes	31 (20.9%)	12 (4.1%)	6.27 (3.11,12.6) **	8.3(3.32,20.6) **
No	117 (79.1%)	284 (95.9%)	1.0	1.0
**History of parental asthma in the family**				
Yes	38 (25.7%)	26 (8.8%)	3.59 (2.08,6.19) **	4.9(2.42,10.18) **
No	110 (74.3%)	270 (91.2%)	1.0	1.0

*AOR *Adjusted Odds Ratio, *COR *Crude Odds Ratio

*Statistically significant at *p* < 0.05, **statistically significant at *p* < 0.01

Others^**^ = refers to private employee, NGO employee and daily laborer

## Discussion

The study was aimed at identifying determinants of community-acquired pneumonia among under-five children in Northeast Ethiopia. Children having older age mother, having mothers who are housewife, not having separate kitchen, having a history of diarrhea in the last 2 weeks, having a history of acute lower respiratory infection in the last 2 weeks and having a history of parental asthma in the family were found to be determinants of community-acquired pneumonia.

Children born from the younger mother were less likely to develop community-acquired pneumonia. A case-control study done in Kersa district, Southwest Ethiopia supports this study [23]. The possible explanation for this might be due to younger women might be more educated, might recognize upper respiratory tract infections earlier and prevent the development of pneumonia. Additionally, younger age mothers might have an opportunity to care for their children with their peer groups than the oldest mothers, which could lead to preventive action for the occurrence of community-acquired pneumonia. Moreover, younger women might use zinc supplementation better than the older one. However, case-control studies conducted in Thailand, Brazil and Southeast Asian countries contradict the current study [10, 31]. The variation of the results could be due to different maternal age categories that different studies used based on their contexts. It might also be explained by the difference in sample size. The sample size of the current is high compared to the above studies. The other possible reason for this difference might be the use of different screening criteria for pneumonia among under-two months up to five years of age children. Screening criteria for pneumonia in this study were integrated management of newborn and child illness guideline, but pneumonia was diagnosed by using clinical presentation and X-ray in Bangladesh, Southern Brazil and Brasilia [21–23].

Children born from mothers who were government employees were less likely to develop childhood community-acquired pneumonia. This finding agreed with the studies done in India, Baghdad/Iraq [10, 12]. This might be because the mothers spend less or no time cooking food for their family while carrying their children on their backs. Thus, reducing their children’s exposure to indoor air pollution is needed. Furthermore, mothers working in professional or technical occupations are likely to be educated. This might also indirectly contribute to their ability to protect their children from infectious diseases such as community-acquired pneumonia.

Children from those households who had no separate kitchen for cooking were more likely to develop childhood community-acquired pneumonia. This finding is similar to a study conducted in Este town, Northwest Ethiopia, Nigeria, Zdola, Zambia, and Kenya [19–21, 26]. The possible reason for this might be in households where there is no separate kitchen for cooking, there could be a high probability of being exposed to air pollutants like fine particles, carbon monoxide, sulfur dioxide, nitrogen dioxide, radon and the likes which could expose children to upper respiratory tract infections including pneumonia. In addition, in such households, there would be poor ventilation that could exacerbate the health risks posed by indoor air pollutants.

Children who had a positive history of diarrhea in the past fifteen days prior to the study were more likely to develop community-acquired pneumonia. Similar studies conducted in urban areas of Oromia Zone, Amhara Region, Tigray, Ethiopia and Iraq, Zimbabwe [9, 25, 26] reported different results with current findings, the difference might be due to variation in methods. This can also be explained by the fact that children who have a concomitant illness like diarrhea may have a lowered immunity, making them more susceptible to diseases like pneumonia.

Children from households with a positive history of acute lower respiratory infection within the past fifteen days prior to the study were more likely to develop community-acquired pneumonia. It is consistent with a study conducted at Kemissie, Ethiopia [9]. The possible explanation might be lower respiratory tract infections are contagious and easily transmitted from household contacts to children [29].

Children from households with a positive history of asthma were more likely to develop community-acquired pneumonia. This result is supported by a case-control study carried out in India [26]. Another similar study reported that maternal history of asthma can increase the risk of severe lower respiratory tract infections in the first year of life [6]. This might be due to respiratory tract infections are easily transmitted from household contacts to children.

Overall, the study findings imply that in Ethiopia, educating women to recognize upper respiratory tract infections earlier to prevent the development of pneumonia and creating awareness about the effect of indoor air pollution to limit the progression of respiratory tract infections to pneumonia would be very important.

The unmatched case control nature of the study did not enable us to control several confounding factors at the same time and during performing logistic regression we might end up with empty strata i.e.; no cases or no control in some strata. The other limitation related to the case control study would be recall bias. Diagnosis of pneumonia was based on clinical WHO IMNCI classification guideline, which could introduce misclassification bias. In addition, the institution-based nature of the study could limit the generalizability of the findings.

## Conclusions

Children having older age mother, having mothers who are housewife, not having separate kitchen, having history of diarrhea in the last 2 weeks, having history of acute lower respiratory infection in the last 2 weeks and having history of parental asthma in the family were found to be determinants of community acquired pneumonia among 2–59 months of age children. Therefore, all health institutions should promote early treatments and prevention of diarrhea and acute lower respiratory infections of children in the health facility and at household level.

## Data Availability

The datasets used and/or analysed during the current study are available from the corresponding author on reasonable request.

## Abbreviations

CAP: Community Acquired Pneumonia
URTI: Upper Respiratory Tract Infection
HMIS: Health Management Information System
IMNCI: Integrated Management of Neonatal and Childhood illness
ARI: Acute Respiratory Tract Infection
